# Association Between Sarcopenic Obesity–Related Scores and Liver Fibrosis in Patients with Steatotic Liver Disease: A Cross-Sectional Study

**DOI:** 10.3390/diagnostics16020324

**Published:** 2026-01-19

**Authors:** Tatsuki Ichikawa, Satoshi Miuma, Mio Yamashima, Shinobu Yamamichi, Makiko Koike, Yusuke Nakano, Hiroyuki Yajima, Osamu Miyazaki, Tomonari Ikeda, Takuma Okamura, Naohiro Komatsu, Mayuko Kakizoe, Ryusei Tanaka, Hisamitsu Miyaaki

**Affiliations:** 1 Department of Gastroenterology, Nagasaki Harbor Medical Center, Nagasaki 850-8555, Japan; 2 Department of Comprehensive Community Care Systems, Graduate School of Biomedical Sciences, Nagasaki University, Nagasaki 852-8501, Japan; 3 Department of Gastroenterology and Hepatology, Graduate School of Biomedical Sciences, Nagasaki University, Nagasaki 852-8501, Japan; 4 Innovation and Translational Research Center, Nagasaki Harbor Medical Center, Nagasaki 850-8555, Japan

**Keywords:** grip strength, obesity, liver stiffness, sarcopenic obesity, difference eGFR

## Abstract

**Background/Objectives**: Sarcopenia (Sp) and obesity (Ob) have significant negative effects on steatotic liver disease (SLD). Here, we examined the effects of sarcopenic Ob (SO) on liver fibrosis in patients with SLD. **Methods:** We included 811 patients who visited our outpatient clinic and underwent FibroScan (Echosens, France). Liver stiffness (LS) was assessed using body mass index (BMI) and grip strength (GS). We conducted a similar analysis by converting the difference in estimated glomerular filtration rate (dGFR) based on creatinine and cystatin C levels into GS. **Results:** The cutoff values for distinguishing metabolic dysfunction-associated steatotic liver disease (MASLD; 298 patients) with LS > 10 kPa (advanced fibrosis) were set separately for men and women using receiver operating characteristic analysis. BMI was set at >26 kg/m^2^ in women and >27 kg/m^2^ in men (modified Ob (mOb)), and GS was set at <16 kg in women and <31 kg in men (modified Sp (mSp)). The ratio of advanced fibrosis was higher in the group with both mSp and mOb (mSpOb) than in the group with mSp alone or mOb alone in MASLD or alcoholic liver disease (ALD, 97 patients). However, this association has not yet been observed in other diseases. The dGFR was used to set the cutoff value corresponding to advanced fibrosis. Sp-dGFR (SpdG) was >1.14 in women and >−0.76 in men in the MASLD group. mSpOb, SpdG and Ob are associated with advanced fibrosis in MASLD logistic regression analysis. **Conclusions:** SO, assessed using BMI and GS or dGFR, was associated with elevated LS in patients with SLD.

## 1. Introduction

The incidence of liver cirrhosis associated with metabolic dysfunction-associated steatotic liver disease (MASLD) has significantly increased worldwide in the past decade [1]. Globally, liver cirrhosis deaths are not declining and are projected to remain among the top 20 causes of death by 2050 [2]. In the Asia-Pacific region, the increasing trend of MASLD is predicted to continue until 2040 [3]. Early detection of MASLD and associated cirrhosis is essential to reduce the global burden of cirrhosis [1].

Recently, the diagnostic criteria for steatotic liver disease have been renewed, and MASLD, alcoholic liver disease (ALD), metabolic dysfunction, and ALD (MetALD) have been defined. A new category, MetALD, was selected to describe those with MASLD, who consume greater amounts of alcohol per week (140–350 g/week and 210–420 g/week for females and males, respectively) [4]. Obesity (Ob), a cardiometabolic risk factor (CMRFs), is a risk factor for advanced fibrosis in patients with MASLD [5]. It has been reported that patients with type 2 diabetes and body mass index (BMI) > 30 kg/m^2^ often have advanced fibrosis (liver stiffness (LS) > 10 kPa) [6]. Sarcopenia (Sp) has also been reported to be an exacerbating factor of liver fibrosis in MASLD [7,8,9]. In cases of MASLD, muscle mass is associated with improved quality of life (QoL), whereas visceral fat mass is associated with worsening QoL [10]. In recent years, sarcopenic Ob (SO), which combines Sp and Ob, has attracted attention as a risk factor for complications of chronic inflammatory diseases, such as cardiovascular disease and diabetes [11]. It has also been reported that the prognosis of solid tumors with SO is worse than that of solid tumors with Ob alone [12]. Therefore, the diagnostic criteria for SO have been established [13,14]. Notably, the criteria for Sp in SO are high body weight and fat mass; therefore, even if there is no absolute loss of muscle mass, relative loss can have clinical and functional consequences [13]. Sarcopenic obesity (SO) is closely associated with myosteatosis and cannot be evaluated using muscle mass alone [15]. It has been reported that SO is associated with cardiovascular risk factors in MASLD but not with liver fibrosis [16]. However, the relationship between MASLD and SO remains unclear.

In this study, we compared BMI, grip strength (GS), and LS to examine the significance of SO in chronic liver disease (CLD). We examined the relationship between LS > 10 kPa, an indicator of advanced fibrosis [17,18,19], and the combination of BMI and GS. In addition, a comparison was made between SO and non-invasive liver fibrosis tests (NITs) (aspartate aminotransferase to platelet ratio index (APRI) [20], fibrosis (FIB)-3 [21], and FIB-4 [22]) for the diagnosis of advanced fibrosis. Markers calculated using serum creatinine and cystatin C levels (Sp index (SI) [23], calculated body muscle mass (cBMM) [24], and estimated glomerular filtration rate (eGFR) (dGFR) [25] are recognized as indicators for estimating Sp. We examined the relationship between these markers, BMI, and advanced fibrosis. The purpose of this study was to examine the relationship between advanced fibrosis, GS, and Ob, then to find a surrogate index for GS, and finally to compare obesity and grip strength (or its surrogate index) with existing NIT.

## 2. Materials and Methods

### 2.1. Patients

This study included 811 patients with CLD who visited our hepatology outpatient clinic between April 2019 and July 2025 for the first time in 2374 patients (Supplementary Figure S1 and Table S1A–C). All patients included in this study were Asian. The exclusion criteria are as follows: returning patients (1543 patients), poorly controlled type 2 diabetes mellitus (10), thyroid dysfunction (5), hypogonadism (0), postmenopausal status without hormonal correction (0), chronic hypercortisolism (0), stage 4 and 5 chronic kidney disease (5), chronic systemic inflammatory (0), extrahepatic malignancies (0), long-term systemic corticosteroids (0), immunosuppressive therapies (0), androgen deprivation therapy (0), incretin agents (0), severe malnutrition (0), malabsorption syndromes (0), prolonged immobilization (0), and neurological disorders (0). The median age (first to third quartiles) was 65 (54–73). A total of 369 women were included in the study. Patients positive for anti-hepatitis C virus antibody (HCV-RNA-positive) or hepatitis B surface antigen were diagnosed with hepatitis C virus (HCV, 82 patients) and hepatitis B virus (HBV, 133 patients), respectively. Primary biliary cholangitis (PBC, 50 patients) and autoimmune hepatitis (AIH, 23 patients) were diagnosed according to the previously reported criteria [26,27]. The diagnostic criteria for MASLD (279 patients), MetALD (19 patients), and ALD (97 patients) were established as previously described. In this report, MetALD was included in MASLD. We retrospectively reviewed the medical records of 811 patients. Clinical data were retrospectively abstracted from patient medical records.

### 2.2. Laboratory Measurements

The difference in GFR (dGFR) was calculated as follows [25]: Cr-based eGFR − CysC-based eGFR. The Sp index (SI) was calculated as [23] Cr/CysC × 100. Body muscle mass (cBMM) was calculated as [body weight (kg) × Cr]/[(K × body weight (kg) × CysC) + Cr] [24], where K = 0.00675 for men and K = 0.01006 for women. The cutoff CBMM values for Sp were 27.903 in women and 39.731 in men [28]. The cutoff of the SARC-F score for Sp was ≥4 points [29]. Grip strength (GS) was measured using a dynamometer (Smedlay Dynamo Meter; TTM, Tokyo, Japan) with participants standing in an erect position with both arms at their sides. The best results of the two tests were used for further analyses. Using the JSH criteria, women with a maximum GS of <18 kg and men with a maximum GS of <28 kg were categorized into the low GS group [30].

Liver stiffness (LS) (kPa) and controlled attenuation parameter (CAP) (dB/m) were measured using FibroScan (Echosens, Paris, France). This FibroScan measurement was possible in all cases; probe type M was used for BMI 30 kg/m^2^ or less, and XL for BMI 30 kg/m^2^ or more, and measurements were taken 10 times. Non-invasive tests (NITs) using blood tests included the following: APRI [20], FIB-3 [21], FIB-4 [22,31], and Child–Pugh scores [32].

All included patients underwent standardized laboratory tests as part of the study protocol.

We created the Definition of Sarcopenia–Obesity–Related Scoring Systems.

1. Sarcopenia and obesity (SpOb): Sarcopenia (Sp) was defined as low GS, specifically <18 kg in women and <28 kg in men. Ob was defined as a BMI ≥ 25 kg/m^2^ for both sexes. Participants were classified into four groups: low GS with Ob (SpOb), Obesity without sarcopenia (Ob), low GS without Ob (Sp), and normal (*N*). The SpOb score was assigned as follows: 0 for N, 1 for Sp or Ob without SpOb, and 2 for SpOb. 2. Modified SpOb (mSpOb): Sarcopenia (Sp) was defined as low GS (<16 kg in women and <31 kg in men), and Ob was defined as high BMI (>26 kg/m^2^ in women and >27 kg/m^2^ in men). Participants were categorized into four groups: low GS with Ob (mSpOb), Obesity without sarcopenia (mOb), low GS without Ob(mSp), and N. The mSpOb score was defined as 0 for N, 1 for mSp or mOb without mSpOb, and 2 for mSpOb. 3. Sarcopenia based on the dGFR and Ob (dGOb): Sarcopenia (Sp) based on the dGFR(SpdG) was defined as high dGFR (>1.14 in women and >−0.76 in men), while Ob was defined as high BMI (>26 kg/m^2^ in women and >27 kg/m^2^ in men). Participants were categorized into four groups: high dGFR with Ob (SpdGOb), Ob without high SpdG (mOb), high SpdG without Ob (SpdG), and N. The dGOb score was defined as 0 for N, 1 for SpdG or mOb without SpdGOb, and 2 for SpdGOb. 4. Sarcopenia based on SI and Ob (SIOb): Sarcopenia (Sp) based on SI (SpSI) was defined as low SI (<67 in women and <84.8 in men), and obesity was defined as high BMI (>26 kg/m^2^ in women and >27 kg/m^2^ in men). Participants were classified into four groups: low SI with Ob (SpSIOb), Ob without high SpSI (mOb), low SI without Ob (SpSI), and N. The SIOb score was assigned as 0 for N, 1 for SpSI or mOb without SpSIOb, and 2 for SpSIOb.

### 2.3. Computed Tomography (CT) Analysis of Body Composition

Cross-sectional CT images of the third lumbar vertebrae were analyzed using Slice-O-Matic version 5.0 (Tomovision, Montreal, QC, Canada) to determine the skeletal muscle (SM) mass. The muscle areas of interest included the psoas, erector spinae, quadratus lumborum, transversus abdominis, external and internal obliques, and rectus abdominis. Tissue Hounsfield unit (HU) thresholds ranging from 29 to 150 HU [33] were used for the SMs. The SMs were normalized to height in m^2^ and expressed as cm^2^/m^2^ to determine the SM index (SMI). Patients with SMI < 39 cm^2^/m^2^ for women and <42 cm^2^/m^2^ for men were categorized into the low SMI group. Sp was diagnosed as a low GS or SMI based on the JSH guidelines for Sp [34].

### 2.4. Statistical Analysis

Data were analyzed using StatFlex (version 6.0; Artech, Osaka, Japan) and are presented as medians and 95% confidence intervals. Laboratory variables were compared using the Mann–Whitney U-tests (for differences between the two groups), analysis of variance (ANOVA, for differences between ≥3 groups), Dunn test (intra-group comparison), and χ^2^ tests. The detection level was analyzed using receiver operating characteristic (ROC) curves. Multivariate analyses were performed using a logistic regression. Correlations were evaluated using Pearson’s correlation coefficient (R). Statistical significance was set at *p* < 0.05.

## 3. Results

In all patients, an association was observed between LS > 10 kPa (advanced fibrosis) and low GS and Ob (SO); however, this association was present only in the MASLD group.

**Figure 1:**
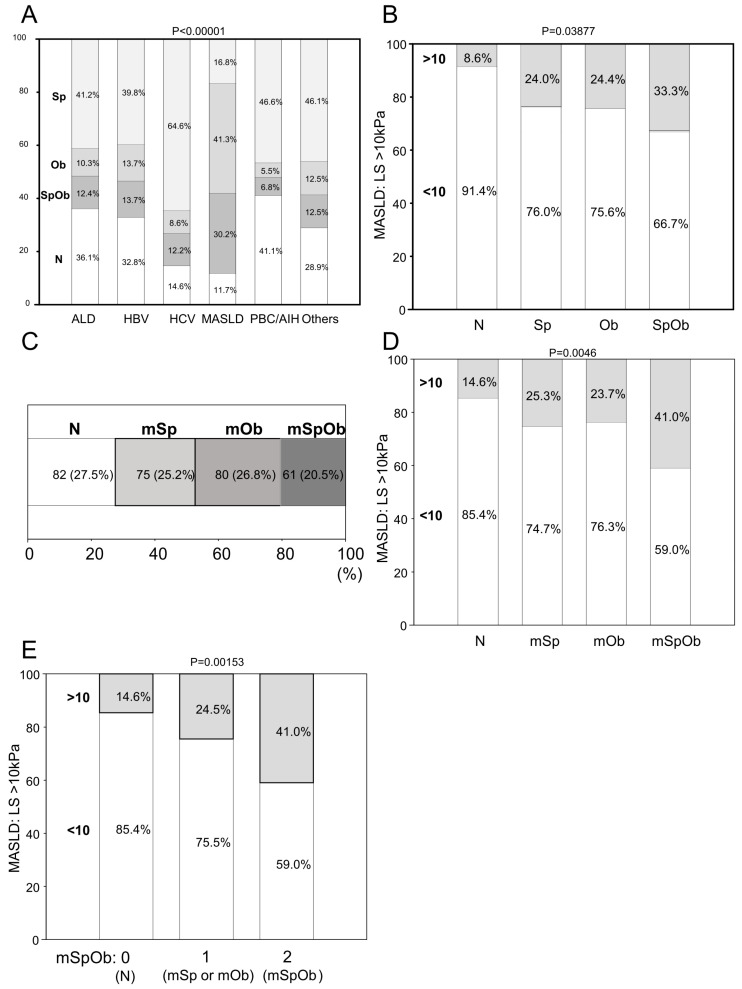
Sarcopenic obesity (SpOb) and modified SpOb in liver disease. (**A**) Distribution of low grip strength and high body mass index in chronic liver disease. Low grip strength (Sp) was defined as <18 kg of mean grip strength in women and <28 kg in men. High body mass index (Ob) was defined as >25 kg/m^2^ in both sexes. Sarcopenic obesity (SpOb) was combined with Sp and Ob. Sp and Ob excluded SpOb. Normal (*N*) did not correspond to either Sp or Ob. ALD is alcoholic liver disease. HBV is an HBV-related disease. HCV is HCV-related disease. MASLD is metabolic dysfunction, steatotic liver disease, metabolic dysfunction, and alcoholic liver (MetALD). *Y*-axis is percentage. *p* value is the result of the chi-square test. (**B**) In MASLD, ratio of liver stiffness (LS) > 10 kPa in each stage (N, Sp, Ob, and SpOb). Ratio of LS > 10 (advanced fibrosis) kPa was the gray area. (**C**) Distribution of modified Sp (mSp), mOb, and mSpOb in MASLD. mSp was defined as <16 kg of mean grip strength in women and <34 kg in men. mOb was defined as >26 kg/m^2^ in women and >27 kg/m^2^ in men. mSpOb was affected by mSp and mOb. mSp and mOb excluded mSpOb. *N* did not correspond to either mSp or mOb. (**D**) In MASLD, ratio of advanced fibrosis kPa in each stage (N, mSp, mOb, and mSpOb). Ratio of advanced fibrosis kPa was the gray area. (**E**) In MASLD, ratio of advanced fibrosis in each mSpOb score. mSpOb score 0 was N, 1 was Sp and Ob without SpOb, and 2 was SpOb.

There were 189 cases with an LS of 10 kPa or above and 622 cases below this value (<10 kPa) in all patients. In the group with advanced fibrosis, BMI was high, and GS was low (Supplementary Table S2A). The group with an advanced fibrosis comprised more men, were older, and had higher CysC (CysCGFR) levels (Supplementary Table S2B). Furthermore, dGFR and strength, assistance walking, rising from a chair, climbing stairs, and falls score (SARC-F) were elevated in the advanced fibrosis group, whereas the SI was low. The prevalence of CBMM-Sp and SARC-F Sp was also higher in the advanced fibrosis group (Supplementary Table S2C). The proportion of SpOb groups varied between diseases (Figure 1A), and only the MASLD group had a higher proportion of SpOb in the LS > 10 kPa group (Figure 1B and Table 1A).

In the mSpOb classification using corrected GS and corrected BMI, an association was observed between advanced fibrosis and mSpOb in MASLD and ALD.

To identify advanced fibrosis, BMI and GS cutoff values were determined according to sex in MASLD. LS (kPa) and BMI (kg/m^2^) were correlated in men (Supplementary Figure S2A,B), whereas GS (kg) was correlated in women (Supplementary Figure S2C,D). In the ROC analysis, the BMI cutoff values for distinguishing advanced fibrosis were 26.6 kg/m^2^ for women and 27.8 kg/m^2^ for men, while the GS cutoff values were 16 kg for women and 31.5 kg for men (Supplementary Figure S2E,F). Women with BMI > 26 kg/m^2^ and men with BMI > 27 kg/m^2^ were classified into the high-BMI group. Women with a GS < 16 kg and men with a GS < 31 kg were classified in the low-GS group. Logistic regression analysis revealed that high BMI and low GS were independent factors contributing to advanced fibrosis (Supplementary Figure S2G). The group with high BMI but not low GS was defined as the modified Ob (mOb); the group with low GS but not high BMI was defined as the modified Sp (mSp); the group with both high BMI and low GS was defined as the modified SpOb (mSpOb); and the group with both normal BMI and GS was defined as the *N* group (Figure 1C). In the MASLD group, advanced fibrosis was more common in the mSpOb than in the mSp, mOb, or *N* group (Figure 1D). When scoring mSp as one point, mOb as one point, and mSpOb as two points (mSpOb score), an increase in the score led to an increase in the proportion of patients with advanced fibrosis in the MASLD group (Figure 1E). A correlation between the mSpOb score and advanced fibrosis was also noted in the ALD group (Table 1B).

dGFR and SI serve as surrogates for Sp, and their combination with mOb was found to correlate with advanced fibrosis in MASLD and ALD.

**Figure 2:**
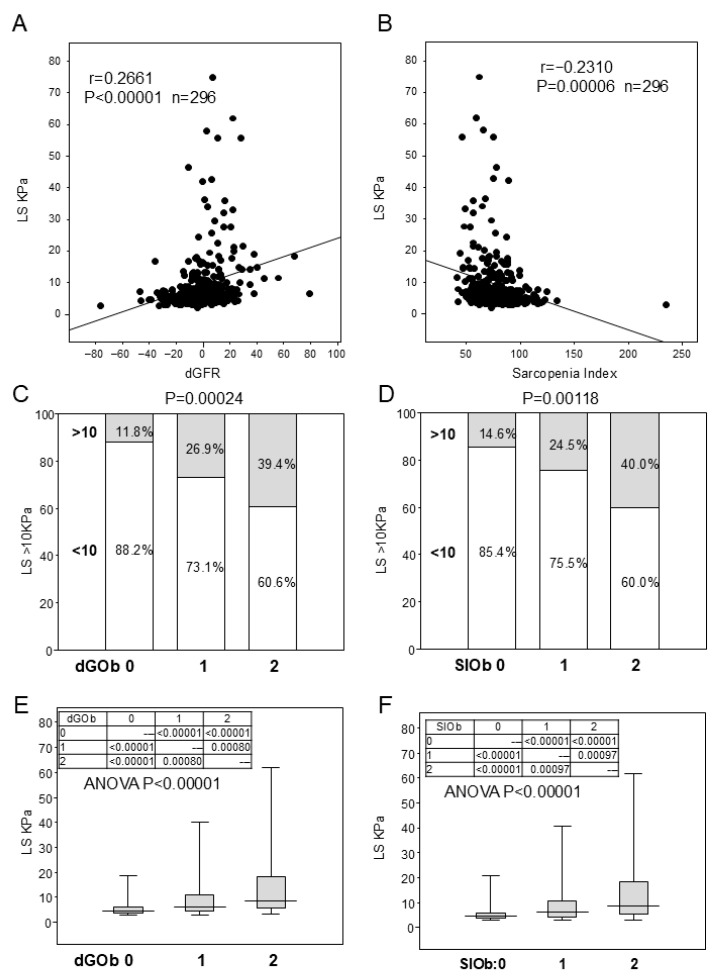
The relation with dGFR, Sarcopenia index, dGOb score, SI score, and liver stiffness in MALSD. (**A**) The relation with liver stiffness (LS) and dGFR. R is correlation factor. *Y* axis is LS (kPa). *X* axis is dGFR. (**B**) The relation with LS and sarcopenia index. *Y* axis is LS (kPa). *X* axis is sarcopenia index. (**C**) Ratio of LS > 10 kPa in each dGOb score. dGFR-mOb score (dGOb score) was calculated as follows: score 0 was normal dGFR and BMI, score 1 is >1.14 of dGFR in women and >−0.76 of dGFR in men (SpdG) without mOb, or mOb (advanced fibrosis) without SpdG, and score 2 is SpdG and mOb. *Y* axis is LS (kPa). *p* value is the result of the chi-square test. (**D**) Ratio of advanced fibrosis in each SIOb score. Sarcopenia index-mOb score (SIOb) was calculated as follows: score 0 was normal SI and BMI, score 1 is <67 of SI in women and <84.8 of SI in men (SpSI) without mOb, or mOb without SpSI, and score 2 is SpSI and mOb. *Y* axis is LS (kPa). *p* value is the result of the chi-square test. (**E**) Value of LS in each dGOb score. Comparisons between the three groups were performed using ANOVA, and comparisons between two groups were performed using the Dunn test. *Y* axis is LS (kPa). (**F**) Value of LS in each SIOb score.

Next, we searched for an indicator to replace GS in MASLD. SI, CBMM, dGFR, and SARC-F were correlated with GS (Table 2A), but only SI and dGFR were correlated with LS (Table 2B and Figure 2A,B). Similarly, low GS (women with GS < 16 kg and men with GS < 31 kg) showed significant differences in SI, CBMM, dGFR, and SARC-F, whereas advanced fibrosis showed significant differences only in dGFR and SI (Table 2B). Based on these results, we determined the cutoff value for distinguishing advanced fibrosis from SI and dGFR using ROC analysis. For women, the dGFR cutoff value was 1.14 and the SI was 67 (Supplementary Figure S3A); for men, the dGFR cutoff value was −0.76 and the SI was 84.8 (Supplementary Figure S3B). SpdGOb was defined as meeting both high dGFR and BMI, whereas *N* was defined as meeting neither of these criteria (Supplementary Figure S3C). The proportion of advanced fibrosis increased in the following order: N, mOb, SpdG, and SpdGOb (Supplementary Figure S3D), and differences in the LS values were also observed (Supplementary Figure S3E). As the dGOb score increased, the proportion of patients with an advanced fibrosis increased (Figure 2C), and the LS (kPa) value also increased (Figure 2E). When SI was scored and examined in the same manner as dGFR (Figure 2D,F), the results for SIOB were identical to those for dGOb. In all patients, dGFR was high, and SI was low in the advanced fibrosis group (Supplementary Table S2C). Therefore, we examined the relationship between dGOb and advanced fibrosis for each disease. ALD was also associated with advanced fibrosis in the dGOb score, similar to MASLD (Table 1C and Supplementary Figure S3F).

Combining mOb with mSp or SpdG associates with high LS in MALSD.

**Figure 3:**
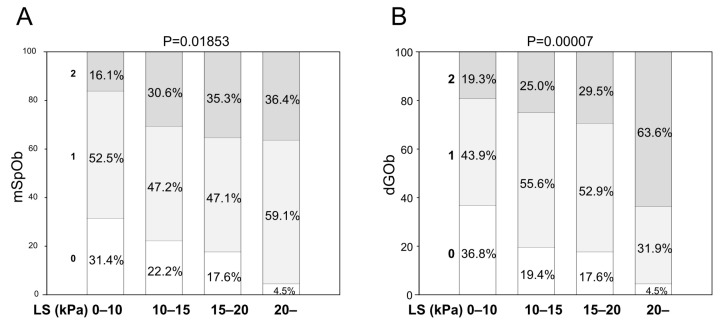
Changes in LS related to mSpOb and dGOb score. (**A**) Changes in LS and mSpOb score. *Y* axis is the proportion of mSpOb score. *X* axis is LS grade. (**B**) dGOb score. *Y* axis is the proportion of dGOb score. *X* axis is LS grade. *p* value is the result of chi-square test.

To investigate the association between elevated LS and sarcopenic Ob (SO), LS was categorized into 0–10, 10–15, 15–20, and >20 kPa, and the relationship between mSpOb (Figure 3A) and dGOb (Figure 3B) scores was examined using the MASLD. Both the mSpOb and dGOb scores showed a prevalence of 0 at low LS levels and an increase of 2 at high LS levels (Supplementary Table S3). The BMI and GS alone were not associated with LS. dGFR alone also showed an inversion in the proportion of SpdG between the LS 10–15 and 15–20 groups, but this proportion was corrected when combined with BMI (Supplementary Table S3).

SO-related scores correlate with advanced fibrosis in the same manner as NITs.

Using multivariate logistic analysis, we compared whether the SO-related scores created in this study (SpOb, mSpOb, dGOb, and SIOb) and non-invasive tests (NITs) (APRI, FIB-3, and FIB-4) were associated with advanced fibrosis (Supplementary Table S4). Sarcopenic obesity (SO)-related scores, such as the FIB-3 and APRI, are associated with advanced fibrosis kPa. High BMI (>26 in women and >27 in men), low GS (<16 kg in women and <31 in men), and low SI (<67 in women and <84 in men) did not individually associate with advanced fibrosis, whereas high dGFR (>1.14 in women and >−0.76 in men) was an independent contributing factor. In contrast, SO-related scores of 2 points were a factor contributing to advanced fibrosis with a statistically significant difference (Table 3A). When SO-related scores of 1 point were used as the cutoff value for advanced fibrosis classification, the sensitivity ranged from 96 to 82.7% and the negative predictive value ranged from 91.4 to 85.3% (Table 3B).

There is no difference in the proportion of advanced fibrosis for the same SO-related score between MASLD and ALD.

To investigate the association between the SO-related scores and ALD, we used sex, age, total bilirubin, albumin, PT-INR, platelet count, AST, ALT, and LS as propensity scores to match the ALD and MASLD groups (Supplementary Table S5). The ALD group had lower Ob rates (Supplementary Figure S4A) and higher prevalence of mSp (Supplementary Figure S4B). SO (mSpOb score 2 and dGOb score 2) were less prevalent in the ALD group (Supplementary Figure S4C,D). In the ALD group, an increase in SO-related scores was associated with a higher proportion of advanced fibrosis (Supplementary Figure S4E (mSpOb) and G (dGOb)). At the same score level, the proportion of LS10 was identical between the ALD and MASLD groups (Supplementary Figure S4F (mSpOb) and H (dGOb)).

In women, an association was observed between SO-related scores and increased VAT, SAT, and SMI in MASLD.

Finally, we compared the SO-related scores and body composition in MASLD cases in which CT was performed (Supplementary Table S6A). Among women, an increase in the mSpOb score was associated with higher VAT and SMI (Supplementary Table S6B), whereas an increase in the dGOb score was associated with higher SAT and SMI (Supplementary Table S6C). No significant differences in VAT (Supplementary Figure S5A) or SMI (Supplementary Figure S5B) were observed between the mSpOb scores of men and women. There was a change in VAT, SAT, and SMI among women; scores 1 and 2 showed greater VAT than scores 0, but there was no difference between scores 1 and 2 (Supplementary Figure S5C–E). Additionally, in women, MA was lower in the mSpOb group, indicating that MA was associated with mSpOb (Supplementary Table S7).

## 4. Discussion

In CLD, the proportion of SpOb varies by disease, and in MASLD, SpOb is associated with advanced fibrosis. In the MASLD group, a low GS (women < 16 kg and men < 31 kg) and high BMI (women > 26 kg/m^2^ and men > 27 kg/m^2^) showed an additive relationship with advanced fibrosis. dGFR and SI were recognized as surrogate indicators for GS, and high dGFR (women with >1.14 and men with >−0.76) and low SI (women with <67 and men with <84.8) were associated with advanced fibrosis in an additive manner with high BMI. Elevated LS was associated with SO-related scores (mSpOb: 2; dGOb: 2). High BMI, GS, and SI did not individually associate with advanced fibrosis, whereas high dGFR was an independent contributing factor. Sarcopenic obesity (SO)-related scores were as significant as NIT in contributing to advanced fibrosis. In the ALD and MASLD groups, the SO-related score was associated with advanced fibrosis. In cases of MASLD, in which body composition analysis was performed, an association was demonstrated between the dGOb score and increases in VAT, SAT, and SMI. We concluded that SO-related scores correlated with LS in patients with MASLD and ALD.

In this study, Sp was assessed using GS as an indicator, and Ob was determined using BMI. SpOb correlates with advanced fibrosis. The cutoff values for GS in the Sp classification were <16 kg for women and <31 kg for men, differing slightly from the Sp criteria (<18 kg for women and <28 kg for men) [30] for both sexes. In addition, the BMI criteria for Ob were set at >26 kg/m^2^ for women and >27 kg/m^2^ for men, a threshold lower than 30 BMI [6]. In studies examining the clinical significance of SO in patients with MASLD, the SO criteria are defined as appendicular skeletal muscle mass (ASM)/body weight (%ASM), with thresholds set at <30.8% for men and <24.3% for women [16]. Furthermore, Ob was defined as fat mass/body weight, set at ≥25% for men and ≥38% for women [16]. This study used dual-energy X-ray absorptiometry (DXA) for body composition analysis; however, unlike in our investigation, it did not identify any association with hepatic fibrosis [16]. In this study, we assessed SO using a simple method based solely on body weight and GS. This method is not only straightforward but has also been shown to correlate with hepatic fibrosis, making it a highly useful approach for determining advanced fibrosis in MASLD. mSpOb is not only associated with advanced fibrosis but may also correlate with elevated LS, making it a highly significant indicator of disease severity in MASLD cases. Whether our SO-related scores fully correspond to the previously proposed SO diagnostic criteria [13,14] has not yet been examined. In some cases, body composition analysis using CT has shown that women with a dGOb of 2 points have higher levels of VAT and SAT, which is not contradictory. However, an increase in SMI was noted in women, which is contradictory. It has been reported that in MASLD, high muscle fat content alongside low skeletal muscle mass constitutes a prognostic factor [35]. Consequently, it has been suggested that incorporating muscle fat assessment in the diagnosis of Sp is more appropriate [36]. Our analysis suggests that muscle fat assessment may have been inadequate, and we hypothesized that this factor is associated with the contradictory results.

In this study, we demonstrated the validity of the dGFR and SI as surrogate indicators of GS. GS measurement is a simple and minimally invasive method that has been implicated in various health disorders [37]. The relationship between GS and prognosis in MASLD is well known [38]. Consideration is also given to the cutoff value of GS in diagnosing SO [39]. SI is a well-known marker of Sp and a prognostic factor; however, in references [23,25,40,41], few reports exist on the relationship between dGFR and Sp [25]. In our previous studies, we reported that, unlike SI, dGFR is not associated with skeletal muscle mass but is linked to GS [25,28] and liver damage [25]. An overestimated Cr-eGFR in patients with cirrhosis is associated with skeletal muscle loss [42]. In recent years, dGFR has been shown to be detrimental to human health [43,44]. It has also been suggested to be associated with Ob; however, as in ref. [45], this cannot be explained solely by reduced creatinine production and decreased CysC clearance [46]. dGFR may serve as an excellent marker for Ob and muscle weakness, and future research should examine its association with chronic liver disease and Ob.

The treatment of MASH has entered an era in which semaglutide [47], a drug proven effective in inducing weight loss, is employed [48]. Among weight-reducing drugs, there is a concern that weight rebound may lead to SO [49,50]. In Ob, weight loss leads to reduced skeletal muscle mass [51]. Repeated weight gain after weight loss exacerbates Sp, which is considered a cause of SO [52]. Weight reduction is important in the treatment of MASLD and MASH; however, SO worsened hepatic fibrosis in our study, and its assessment is considered highly important. A simple and rapid method is required for assessing SO, for which measurements of BMI, GS, Cr, and CysC are considered useful.

This study revealed that SO-related scores were associated with advanced fibrosis in both MASLD and ALD. ALD had lower Ob rates; however, there was no difference in the proportion of patients with advanced fibrosis at the same mSpOb or dGOb scores. Unlike other CLDs, SLD affects SO-induced liver fibrosis. Whether SO is a cause or consequence of increased LS in SLD remains unclear; however, liver dysfunction, Sp, and Ob are interdependent [53]. Monitoring muscle mass while reducing weight is essential, and establishing a simple method for assessing muscle and fat mass is necessary for the treatment of SLD. It is also important to investigate whether mSpOb and dGOb, which we determined to be useful markers for advanced fibrosis, correlate with SLD prognosis.

This study also suggests a relationship between myosteatosis (MA) and the sarcopenic obesity–related factor (mSpOb) in women, indicating that in severely obese patients, muscle strength may be more important to assess than muscle mass. In addition, liver stiffness measurement can be problematic in patients with severe obesity [54], making it necessary to evaluate liver fibrosis using additional NITs. Among these NITs, APRI and FIB-3 were associated with advanced fibrosis independently of sarcopenia–obesity–related factors, suggesting that future comparative studies focusing on severely obese patients will be required.

This study has some limitations. As this was a cross-sectional study conducted in a specialized liver disease facility, there was bias in the cases. Because the number of MetALD cases in this study was limited, they were included within the MASLD group, and analyses were performed by comparing MASLD and ALD. If a sufficient number of MetALD cases becomes available in future studies, a three-group comparison would be more appropriate. In CLDs other than SLD, SO-related scores showed no association with LS; however, as most cases occurred prior to HCV treatment or before nucleoside analog intervention for HBV infection, the state following viral clearance could not be assessed. In this study, patients with CKD stages 4–5 were excluded, whereas those with CKD stage 3 were included. The inclusion of patients with impaired renal function may have influenced the performance of indices based on creatinine, cystatin C, and dGFR. Therefore, future studies should evaluate the usefulness of the SO-related score specifically in patients with CKD stages 1–2. Differences in the number of cases for each disease and in age distribution may influence the observed relationship between myosteatosis and liver stiffness. In this study, validation of the AUC for discriminating advanced fibrosis was not feasible due to the lack of an external cohort. Instead of internal cross-validation, we examined sex-specific differences, and the AUCs appeared relatively stable (Supplementary Figures S2E,F and S3A,B). Validation using an external cohort will be required in future studies. Although MASLD and MetALD are distinct entities, MetALD cases were included in the MASLD group in the present analysis because of their small number. Propensity score-matched comparisons between ALD and MASLD (including MetALD) showed no difference in their association with SO-related score and advanced fibrosis, indicating that further studies focusing on MetALD are needed. Body composition analysis was not performed in all patients, and the criteria for diagnosing SO were not met. Muscle fat (myosteatosis) could not be diagnosed.

In patients with SLD, the combination of low GS and high BMI was associated with advanced fibrosis. Similar results were obtained when SI or dGFR were used instead of GS. SO-related scores demonstrated an association with LS. Although weight loss is important in the treatment of MASH, weight rebound after weight loss can cause SO. Muscle monitoring using GS, Cr, and CysC is important for diagnosing liver fibrosis and follow-up after treatment.

## Figures and Tables

**Table 1:** (**A**) Relationship between SpOb and LS > 10 in each disease. (**B**) Relation between modified SpOb score and LS > 10 kPa in each disease. (**C**) Relation between dGOb score and LS >10 kPa in each disease.

**(A)**																		
**SpOb**	**ALD**			**HBV**			**HCV**			**MASLD**			**PBC/AIH**			**Others**		
**LS**	**<10**	**>10**	***p***	**<10**	**>10**	***p***	**<10**	**>10**	***p***	**<10**	**>10**	***p***	**<10**	**>10**	***p***	**<10**	**>10**	***p***
*N*	22 (44.9)	13 (27.1)	0.10663	40 (34.5)	3 (20.0)	0.61162	8 (12.9)	4 (20.0)	0.76808	32 (14.4)	3 (4.0)	0.03877	27 (48.2)	3 (17.6)	0.10881	34 (29.8)	3 (21.4)	0.26585
SpOb	3 (6.1)	9 (18.8)		15 (12.9)	3 (20.0)		7 (11.3)	3 (15.0)		60 (26.9)	30 (40.0)		3 (5.4)	2 (11.8)		14 (12.3)	2 (14.3)	
Ob	6 (12.3)	4 (8.3)		15 (12.9)	3 (20.0)		5 (8.1)	2 (10.0)		93 (41.7)	30 (40.0)		2 (3.6)	2 (11.8)		12 (10.5)	4 (28.6)	
Sp	18 (36.7)	22 (45.8)		46 (39.7)	6 (40.0)		42 (67.7)	11 (55.0)		38 (17.0)	12 (16.0)		24 (42.8)	10 (58.8)		54 (47.4)	5 (35.7)	
**(B)**																		
**mSpOb**	**ALD**			**HBV**			**HCV**			**MASLD**			**PBC/AIH**			**Others**		
**LS**	**<10**	**>10**	***p***	**<10**	**>10**	***p***	**<10**	**>10**	***p***	**<10**	**>10**	***p***	**<10**	**>10**	***p***	**<10**	**>10**	***p***
0	22 (44.9)	5 (10.4)	0.00016	47 (40.5)	3 (20.0)	0.05520	14 (23.0)	5 (25.0)	0.21292	70 (31.4)	12 (16.0)	0.00153	32 (57.1)	7 (41.2)	0.24773	52 (45.6)	4 (28.6)	0.22234
1	26 (53.1)	35 (72.9)		63 (54.3)	9 (60.0)		46 (75.4)	13 (65.0)		117 (52.5)	38 (50.7)		24 (42.9)	10 (58.8)		55 (48.2)	10 (71.4)	
2	1 (2.0)	8 (16.7)		6 (5.2)	3 (20.0)		1 (1.6)	2 (10.0)		36 (16.1)	25 (33.3)		0 (0.0)	0 (0.0)		7 (6.2)	0 (0.0)	
**(C)**																		
**dGOb**	**ALD**			**HBV**			**HCV**			**MASLD**			**PBC/AIH**			**Others**		
**LS**	**>10**	**<10**	***p***	**>10**	**<10**	***p***	**>10**	**<10**	***p***	**>10**	**<10**	***p***	**>10**	**<10**	***p***	**>10**	**<10**	***p***
0	7 (14.6)	18 (36.7)	0.00547	3 (20.0)	53 (45.3)	0.09374	3 (15.0)	13 (21.0)	0.62564	11 (14.7)	82 (36.8)	0.00024	3 (17.6)	30 (53.6)	0.00995	3 (21.4)	60 (52.6)	0.06592
1	33 (68.7)	30 (61.3)		10 (66.7)	59 (50.4)		15 (75.0)	46 (74.2)		36 (48.0)	98 (43.9)		13 (76.5)	26 (46.4)		10 (71.4)	45 (39.5)	
2	8 (16.7)	1 (2.0)		2 (13.3)	5 (4.3)		2 (10.0)	3 (4.8)		28 (37.3)	43 (19.3)		1 (5.9)	0 (0.0)		1 (7.2)	9 (7.9)	

(**A**) Low grip strength (Sp) was defined as a mean grip strength < 18 kg in women and <28 kg in men. A high body mass index (BMI) was defined as a BMI > 25 kg/m^2^ in both sexes. Sarcopenic obesity (SpOb) was affected by Sp and Ob levels. Sp and Ob, excluding SpOb. Normal (*N*) levels do not correspond to Sp or Ob. *p*-values were calculated using the LXM chi-squared test. (**B**) The mSp was defined as a mean grip strength of <16 kg in women and <31 kg in men. The mOb was defined as >26 kg/m^2^ in women and >27 kg/m^2^ in men. mSpOb is affected by mSp and mOb. mSp and mOb, excluding mSpOb. *N* does not correspond to either mSp or mOb. Sp and Ob were scored as 0, 1, or 2, respectively. *p*-values were calculated using the LXM chi-squared test. (**C**) SpdG was defined as <1.14 of dGFR in women and <−0.76 in men. The mOb was defined as >26 kg/m^2^ in women and >27 kg/m^2^ in men. The SpdGOb includes both SpGFR and mOb. *N* did not correspond to SpdG or mOb. A dGOb score of 1 indicated SpdG without mOb or mOb without SpdG, and a score of 2 indicated both SpdG and mOb. *p*-values were calculated using the LXM chi-squared test.

**Table 2:** (**A**) The relation with GS, LS, and Cr, CysC, and SARC-F in MASLD. (**B**) Low GS (mSp) and LS > 10 kPa were related to SI, CBMM, dGFR, and SARC-F in MASLD.

**(A)**							
		**GS/r**	**GS/P**		**LS/r**	**LS/P**	
Creatinine (mg/mL)		0.3514	<0.00001		−0.0261	0.65404	
Cystatin C (mg/L)		−0.1507	0.00920		0.1886	0.00107	
CrGFR (ml/min/1.73 m^2^)		0.1195	0.03917		0.0487	0.40175	
CysCGFR (ml/min/1.73 m^2^)		0.2700	<0.00001		−0.1839	0.00143	
SI		0.5472	<0.00001		−0.2310	0.00006	
CBMM		0.8016	<0.00001		−0.0671	0.24814	
dGFR		−0.2125	0.00022		0.2661	<0.00001	
SARC-F		−0.4636	<0.00001		0.0486	0.40296	
**(B)**							
		**Low GS 16/31**			**LS > 10 kPa**		
		***N***	**mSp**	***p***	**<10**	**>10**	***p***
SI	*n*	162	136	<0.00001	223	75	0.00064
	Me (Q1~Q3)	79.1 (68.6~91.7)	68.9 (58.6~82.3)		76.9 (65.8~90.4)	70.5 (56.6~81.7)	
CBMM	*n*	162	136	<0.00001	223	75	0.68884
	Me (Q1~Q3)	38.97 (34.01~48.32)	34.48 (30.06~43.17)		36.90 (32.10~45.16)	36.64 (31.35~45.74)	
dGFR	*n*	162	136	<0.00001	223	75	<0.00001
	Me (Q1~Q3)	−4.9 (−15.9~3.5)	3.0 (−4.25~13.15)		−2.70 (−13.95~6.95)	5.00 (−3.62~15.75)	
SARC-F	*n*	162	136	<0.00001	223	75	0.71847
	Me (Q1~Q3)	0.0 (0.0~1.0)	1.0 (0.0~3.0)		1.0 (0.0~2.0)	1.0 (0.0~2.0)	

The abbreviations used are as follows: correlation factor (r), grip strength (GS), liver stiffness (LS), creatinine (Cr), Cystatin C (CysC), sarcopenia index (SI), calculated body muscle mass (CBMM), and differences in CrGFR and CysCGFR. Low GS (modified Sp) was defined as a weight < 16 kg in women and <31 kg in men, where *n* denotes the number of patients. Me (Q1~Q3) is the median (first to third quartiles). *p*-values were calculated using the Mann–Whitney U test. The abbreviations used are as follows: modified Sp (mSp).

**Table 3:** (**A**) Obesity and muscle-related factors contribute additively to LS > 10 kPa in MASLD. (**B**) Evaluation of cut-off values for determining LS10 in non-invasive liver fibrosis test and sarcopenic obesity test in MASLD.

**(A)**							
**Score (Point)**		***p***	**Odds Ratio**		**95% CI Lower**	**95% CI Upper**	
SpOb; 0			1				
1		0.05071	3.420		0.996	11.740	
2		0.00933	2.309		1.229	4.340	
mSpOb; 0			1				
1		0.07916	1.895		0.928	3.867	
2		0.00058	2.013		1.351	2.998	
dGOb; 0			1				
1		0.00732	2.738		1.312	5.718	
2		0.00009	2.203		1.485	3.269	
SIOb; 0			1				
1		0.07561	1.893		0.936	3.827	
2		0.00044	1.974		1.351	2.884	
**(B)**							
**LS10 kPa**		**>10 *N* (%)**	**<10 *N* (%)**	**Sensitivity**	**Specificity**	**PPV**	**NPV**
APRI 1.5	Normal	46 (61.3)	198 (88.8)	38.7	88.8	53.7	81.1
	High	29 (38.7)	25 (11.2)				
FIB-3 1.89	Normal	10 (13.3)	126 (56.5)	86.7	56.5	40.1	92.6
	High	65 (86.7)	97 (43.5)				
SpOb 1	Normal	3 (4.0)	32 (14.3)	96	14.3	27.4	91.4
	High	72 (96.0)	191 (85.7)				
mSpOb 1	Normal	12 (16.0)	70 (31.4)	84	31.4	29.2	85.3
	High	63 (84.0)	153 (68.6)				
SIOb 1	Normal	13 (17.3)	76 (34.1)	82.7	34.1	29.7	85.3
	High	62 (82.7)	147 (65.9)				
dGOb 1	Normal	11 (14.7)	82 (36.8)	85.3	36.8	31.2	88.1
	High	64 (85.3)	141 (63.2)				

Logistic regression analysis was performed to determine whether a single (obesity or muscle-related factor: 1 point) or both obesity- and muscle-related factors (2 points) contributed to LS > 10 kPa. Non-invasive liver fibrosis tests: APRI > 1.5 and FIB-3 > 1.85. Sarcopenic obesity test: SpOb > 1, mSpOb > 1, SIOb > 1, and dGOb > 1. *N* is number. PPV, positive predictive value; NPV, negative predictive value.

## Data Availability

The original contributions presented in this study are included in the article/Supplementary Materials. Further inquiries can be directed to the corresponding author.

## Supplementary Materials

The following supporting information can be downloaded at https://www.mdpi.com/article/10.3390/diagnostics16020324/s1. Supplementary Figure S1, A flow chart outlining patient inclusion, exclusion. Supplementary Figure S2, Setting BMI and GS cutoff values related to LS > 10 kPa (advanced fibrosis) in MASLD. (A) The relation between LS and BMI in women. *Y* axis is LS (kPa). *X* axis is BMI (kg/m^2^). R is the correlation factor. (B) The relation between LS and BMI in men. (C) The relation between LS and GS in women. *X* axis is GS (kg). (D) The relation between LS and GS in men. (E,F) Receiver operating characteristic (ROC) analysis for advanced fibrosis in MALSD. Black line is BMI and red line is GS. The cut-off value was set at a value where sensitivity and specificity were equal. AUC is the area under the curve. The cutoff points were 26.6 of BMI and 16 of GS in women (E) and 27.8 of BMI and 31.5 of GS in men (F). (G) Multivariate logistic regression analysis was performed to analyze whether low grip strength and high BMI, defined by cut-off values, were associated with advanced fibrosis. High BMI (modified Ob; mOb) was >26.6 kg/m^2^ in women and >27.8 kg/m^2^ in men. Low GS (modified Sp; mSp) was <16 kg in women and <31.6 kg in men. mOb and mSp are associated with independent to advanced fibrosis. Supplementary Figure S3. Setting the dGFR and SI cutoff value related to advanced fibrosis in MASLD. (A,B) Receiver operating characteristic (ROC) analysis for advanced fibrosis in MALSD. The black line represents the dGFR, and the red line represents the SI. The cutoff value was set at a value where the sensitivity and specificity were equal. AUC is the area under the curve. The cutoff points were 1.14 of dGFR and 67 of SI in women (A) and −0.76 of dGFR and 84.3 of GS in men (B). (C) Distribution of SpdG, mOb, and SpdGOb in MASLD. SpdG was defined as <1.14 kg of dGFR in women and <−0.76 kg in men without mOb. The mOb was defined as >26 kg/m^2^ in women and >34 kg/m^2^ in men without SpdG. The SpdGOb includes both SpGFR and mOb. *N* did not correspond to either SpGFR or mOb levels. (D) Rate of advanced fibrosis in N, mOb, SpdG, and SpdGOb in MASLD. The *p*-value represents the results of the chi-square test. (E) Value of LS (kPa) in each dGOb score in MASLD. Comparisons between three groups were performed using analysis of variance (ANOVA), and comparisons between two groups were performed using the Dunn test. The *Y*-axis represents the LS (kPa). (F) Value of LS (kPa) in each dGOb score in ALD. Supplementary Figure S4, Comparison between the two groups after matching the propensity scores of ALD and MASLD with sex, age, total bilirubin, albumin, prothrombin time, INR, platelet, AST, ALT, and LS as covariates. (A) Rate of obesity (Ob) (BMI > 26 in women and >27 in men) in ALD and MASLT. *p* value is 0.00058 by chi-square test. (B) Distribution of normal (*N*), mSp, mOb, and mSpOb. mOb is Ob (>26 of BMI in women and >27 in men) without low grip strength (GS) (<16 kg in women and <31 kg in men). mSp is low GS without Ob. mSpOb is both Ob and low GS. Normal (*N*) is either Ob or low GS. *p* value is 0.02129 by chi-square test. (C) Rate of mSpOb score. Score 0 is N, score 1 is mOb or mSp, and score 2 is mSpOb. *p* value is 0.02129 by chi-square test. (D) Rate of dGOb score. Score 0 is N, score 1 is mOb or SpdG, and Score 2 is SpdGOb. *p* value is 0.00125 by chi-square test. (E) Differences in the proportion of advanced fibrosis based on mSpOb score within each disease group. *Y* axis is the rate of advanced fibrosis. *p* value calculated by chi-square test. (F) Differences in the proportion of advanced fibrosis between disease groups with the same mSpOb score. (G) Differences in the proportion of advanced fibrosis based on dGOb score within each disease group. (H) Differences in the proportion of advanced fibrosis between disease groups with the same dGOb score. Supplementary Figure S5, Comparison with body compositions in the score of mSpOb and dGOb. (A) Comparison of VAT by mSpOb score. *Y* axis is VAT (cm^2^). *p* values were calculated using ANOVA for comparisons of VAT within the same sex group. (B) Comparison of SMI by mSpOb score. *Y* axis is SMI (cm^2^/m^2^). (C) Comparison of VAT by dGOb. *Y* axis is VAT (cm^2^). W is women group. When there was a significant difference in ANOVA, we added a Dunn test. The table shows the *p* values obtained by Dunn’s test for each item within the same group. (D) Comparison of SAT by dGOb. *Y* axis is SAT (cm^2^). M is man group. (E) Comparison of SMI by dGOb. *Y* axis is SMI (cm^2^/m^2^). Table S1A. Clinica factors in each disease, Table S1B. Clinical factors related liver reserve and non-invasive liver fibrosis test. Table S1C. Body mass index and muscle related factors. Table S2A. The relation with liver stiffness (LS) >10 kPa and body mass index and grip strength in all patients. Table S2B. The relation with liver stiffness >10 kPa and clinical factors in all patients. Table S2C. The relation with liver stiffness >10 kPa and non-invasive liver fibrosis tests, muscle markers in all patients. Table S3. Progression of LS related to mSpOb and dGOb score. Table S4. SpOb, mSpOb, SIOb and dGOb were contributed to LS > 10 kPa in MASLD. Table S5. Difference between ALD and MASLD after propensity score matched. Table S6A. Background of the group whose body composition was examined by CT in MASLD. Table S6B. Comparison of body composition by mSpOb score in MASLD. Table S6C. Comparison of body composition by dGOb score in MASLD. Table S7. Comparison of body composition between mSpOb and non-mSpOb in MASLD.

## Abbreviations

Alcoholic liver disease; ALD, Body mass index; BMI, chronic liver disease; CLD, calculated body muscle mass; cBMM, controlled attenuation parameter; CAP, Cardio metabolic risk factors; CMRF, creatinine; Cr, cystatin C; CysC, difference eGFR; dGFR, Sarcopenia based on the dGFR and Ob; dGOb, liver stiffness; LS, estimated glomerular filtration rate; eGFR, grip strength; GS, metabolic dysfunction associated steatotic liver disease; MASLD, metabolic dysfunction and ALD; MetALD, non-invasive liver fibrosis tests; NITs, Obesity; Ob, Receiver operating characteristic; ROC, strength, assistance walking, rising from a chair, climbing stairs and falls; SARC-F, sarcopenia; Sp, sarcopenia index; SI, Sarcopenic obesity; SO, Sarcopenia and obesity; SpOb, sarcopenia based on SI and Ob; SIOb.
