# Supplementary material for: Association Between Sarcopenic Obesity–Related Scores and Liver Fibrosis in Patients with Steatotic Liver Disease: A Cross-Sectional Study
Source: Diagnostics (Basel). 2026 Jan 19;16(2):324. doi: 10.3390/diagnostics16020324 (PMC12840360; doi:10.3390/diagnostics16020324)
Supplement: Supplementary file 1 [file diagnostics-16-00324-s001.zip › diagnostics-3999323-supplementary/Supp. table.pdf]

Supplementary Table S1A. Clinica factors in each disease

		ALD (97)	HBV (133)	HCV (82)	MASLD (298)	PBC/AIH (73)	Others (128)	P value
Sex	Women	18 (18.6)	71 (53.4)	42 (51.2)	179 (60.1)	59 (80.8)	73 (57.0)	<0.00001
	Men	79 (81.4)	62 (46.6)	40 (48.8)	119 (39.9)	14 (19.2)	55 (43.0)	
Age	n	97	133	82	298	73	128	<0.00001
	Me(Q1~Q3)	63.0 (54.0~71.3)	66.0 (55.8~75.0)	73.0 (65.0~81.0)	61.0 (51.0~70.0)	67.0 (54.8~75.3)	67.0 (49.0~76.0)	
Total Bilirubin (mg/dl)	n	97	132	82	298	73	128	<0.00001
	Me(Q1~Q3)	1.0 (0.70~1.50)	0.70 (0.50~0.90)	0.70 (0.50~0.90)	0.70 (0.60~1.00)	0.70 (0.50~0.90)	0.80 (0.60~1.10)	
Albumin (g/dl)	n	97	132	82	297	73	128	<0.00001
	Me(Q1~Q3)	4.00 (3.60~4.30)	4.20 (3.80~4.40)	4.00 (3.50~4.20)	4.40 (4.10~4.50)	4.10 (3.88~4.40)	4.20 (3.95~4.50)	
Prothrombin time-INR	n	97	132	82	298	73	128	0.00001
	Me(Q1~Q3)	1.05 (0.95~1.13)	1.00 (0.95~1.05)	1.02 (0.97~1.07)	0.98 (0.94~1.03)	0.99 (0.95~1.04)	1.00 (0.95~1.04)	
Creatinine (Cr)	n	97	132	82	298	73	128	0.00391

(mg/dl)	Me(Q1~Q3)	0.77 (0.68~0.97)	0.79 (0.67~0.92)	0.81 (0.66~0.98)	0.73 (0.63~0.87)	0.71(0.61~0.83)	0.74 (0.64~0.88)	
CrGFR (ml/min/1,73m^2)	n	97	132	82	298	73	128	0.00030
	Me(Q1~Q3)	76.60 (60.48~87.40)	67.60 (55.90~78.30)	64.80 (49.50~78.60)	70.40 (59.80~81.60)	66.40 (58.50~78.50)	70.45 (56.85~84.15)	
CystatinC (CysC) (mg/l)	n	97	132	82	298	73	128	<0.00001
	Me(Q1~Q3)	1.11 (0.925~1.37)	1.015 (0.86~1.265)	1.24 (1.03~1.63)	0.97 (0.85~1.12)	1.02 (0.835~1.3025)	0.955 (0.825~1.185)	
CysCGFR (ml/min/1,73m^2)	n	97	132	82	298	73	128	<0.00001
	Me(Q1~Q3)	64.7 (48.52~80.52)	69.6 (52.3~83.9)	53.1 (35.3~68.1)	72.1 (58.9~86.8)	63.7 (46.4~84.5)	75.9 (52.9~91.5)	
Platelet (X10^4/μl)	n	97	132	82	298	73	128	0.00001
	Me(Q1~Q3)	17.30 (12.40~22.48)	18.30 (14.85~23.40)	18.15 (13.10~22.50)	21.15 (16.90~24.90)	19.50 (15.33~24.40)	20.70 (17.50~24.10)	
AST (U/l)	n	97	132	82	298	73	128	<0.00001
	Me(Q1~Q3	48.0	24.0	34.0	45.0	45.0	30.5	

	)	(28.0~80.0)	(19.0~33.5)	(25.0~65.0)	(31.0~72.0)	(29.8~74.5)	(22.0~56.0)	
ALT (U/l)	n	97	132	82	298	73	128	<0.00001
	Me(Q1~Q3)	34.00 (20.00~67.25)	21.00 (15.00~36.00)	34.50 (19.00~56.00)	61.00 (35.00~96.00)	45.00 (25.75~76.50)	34.00 (20.50~73.50)	

Normal range is the follows; total bilirubin; 0.3-1.2 mg/dl, albumin, albumin; 3.8-5.2 g/dl, prothrombin time - international normalized ration (INR); 1.8-1.2, Creatinine (Cr); 0.47-0.79 mg/dl in woman and 0.61-1.04 mg/dl in man. Cr-estimated glomerular filtration rate (CrGRR) : >60 ml/min/1,73m^2, cystatin C (CysC); 0.56-0.87 mg/l in woman and 0.63-0.95 mg/l in man, CysC-eGFR; >60 ml/min/1,73m^2, platelet; 13-36.9 X10^4/μl in woman and 13.1-36.2 X10^4/μl in man. Aspartate aminotransferase (AST); 10-40 U/l, Alanine aminotransferase (ALT); 5-40 U/l. n is number of patients. Me (Q1-Q3) is median (first quartile to the third quartile). P-values were calculated using the LXM chi-square test or the Kruskal-Wallis test.

Supplementary Table S1B. Clinical factors related liver reserve and non-invasive liver fibrosis test.

		ALD (97)	HBV (133)	HCV (82)	MASLD (298)	PBC/AIH (73)	Others (128)	P value
CPS	n	97	132	82	298	73	128	0.00873
	Me(Q1~Q3)	5.0 (5.0~6.0)	5.0 (5.0~5.0)	5.0 (5.0~6.0)	5.0 (5.0~5.0)	5.0 (5.0~5.0)	5.0 (5.0~5.0)	
CPG	A	80 (82.5)	126 (95.5)	72 (87.8)	291 (97.7)	70 (95.9)	124 (96.9)	0.00001
	B	16 (16.5)	6 (4.5)	10 (12.2)	6 (2.0)	3 (4.1)	3 (2.3)	
	C	1 (1.0)	0 (0.0)	0 (0.0)	1 (0.3)	0 (0.0)	1 (0.8)	

Hepatic encephalopathy	1	93 (95.9)	133 (100.0)	81 (98.8)	298 (100.0)	72 (98.6)	128 (100.0)	0.99418
	2	3 (3.1)	0 (0.0)	1 (1.2)	0 (0.0)	1 (1.4)	0 (0.0)	
	3	1 (1.0)	0 (0.0)	0 (0.0)	0 (0.0)	0 (0.0)	0 (0.0)	
Ascites	1	84 (86.6)	131 (98.5)	78 (95.1)	295 (99.0)	72 (98.6)	125 (97.7)	0.58438
	2	11 (11.3)	2 (1.5)	4 (4.9)	3 (1.0)	1 (1.4)	3 (2.3)	
	3	2 (2.1)	0 (0.0)	0 (0.0)	0 (0.0)	0 (0.0)	0 (0.0)	
MELD	n	97	132	82	298	73	128	<0.00001
	Me(Q1~Q3)	8 (7~10)	6 (6~8)	7 (6~8)	6 (6~7)	6 (6~7)	6 (6~7)	
FIB-4	n	97	132	82	298	73	128	<0.00001
	Me(Q1~Q3)	3.1 (1.8~6.2)	1.8 (1.2~2.7)	2.8 (1.7~4.0)	1.8 (1.1~2.8)	2.3 (1.5~3.9)	1.7 (1.0~2.8)	
ALBI	n	97	132	82	298	73	128	<0.00001
	Me(Q1~Q3)	-2.5626 (-2.8720~-2.2751)	-2.8475 (-3.0406~-2.5319)	-2.6941 (-2.9338~-2.30390)	-2.9589 (-3.1249~-2.7562)	-2.8176 (-2.9848~-2.5246)	-2.8641 (-3.0726~-2.5639)	
ALBIG	1	48 (49.5)	97 (73.5)	50 (61.0)	258 (86.6)	52 (71.2)	92 (71.9)	<0.00001
	2	45 (46.4)	34 (25.8)	31 (37.8)	36 (12.1)	21 (28.8)	32 (25.0)	
	3	4 (4.1)	1 (0.8)	1 (1.2)	4 (1.3)	0 (0.0)	4 (3.1)	
APRI	n	97	132	82	298	73	128	<0.00001
	Me(Q1~Q3)	1.1 (0.5~2.0)	0.4 (0.3~0.7)	0.7 (0.4~1.5)	0.7 (0.5~1.3)	0.7 (0.5~1.5)	0.5 (0.3~1.1)	
FIB-3	n	97	132	82	298	73	128	<0.00001

	Me(Q1~Q3)	4.2 (2.1~6.4)	1.5 (0.3~2.7)	2.7 (1.1~4.4)	2.2 (0.8~4.0)	2.7 (1.4~5.1)	1.5 (0.2~3.8)	
LS (kPa)	n	97	133	82	298	73	128	<0.00001
	Me(Q1~Q3)	9.7 (5.2~21.725)	4.3 (3.4~5.85)	6.4 (4.9~9.8)	6.3 (4.5~10.0)	5.3 (4.0~8.4)	4.3 (3.6~5.95)	
LS>20kPa	LS>20	26 (26.8)	4 (3.0)	6 (7.3)	22 (7.4)	5 (6.8)	4 (3.1)	<0.0001
	LS<20	71 (73.2)	129 (97.0)	76 (92.7)	276 (92.6)	68 (93.2)	124 (96.9)	
LS>10kPa	LS<10	49 (50.5)	118 (88.7)	62 (75.6)	223 (74.8)	56 (76.7)	114 (89.1)	<0.00001
	LS>10	48 (49.5)	15 (11.3)	20 (24.4)	75 (25.2)	17 (23.3)	14 (10.9)	
LS>8kPa	LS<8	46 (47.4)	114 (85.7)	48 (58.5)	197 (66.1)	53 (72.6)	113 (88.3)	<0.00000
	LS>8	51 (52.6)	19 (14.3)	34 (41.5)	101 (33.9)	20 (27.4)	15 (11.7)	
CAP (dB/m)	n	97	133	82	298	73	128	<0.00000
	Me(Q1~Q3)	261.0 (222.8~297.3)	232.0 (203.8~262.3)	214.0 (191.0~241.0)	302.0 (263.0~334.0)	216.0 (188.5~254.0)	222.5 (191.5~255.0)	
CAP>240 dB/m	<240	33 (34.0)	75 (56.4)	61 (74.4)	31 (10.4)	48 (65.8)	79 (61.7)	<0.00000
	>240	64 (66.0)	58 (43.6)	21 (25.6)	267 (89.6)	25 (34.2)	49 (38.3)	

The abbreviations are as follows; Child-Pugh score (CPS), CP grade (CPG), MELD (model for end-stage liver disease), fibrosis-4 index (FIB-4), albumin-bilirubin score (ALBI), ALBI grade (ALBIG), AST to platelet ratio (APRI), fibrosis- 3 index (FIB-3), liver stiffness (LS), controlled attenuation parameter (CAP). Hepatic encephalopathy and ascites are scored as 1, 2, and 3 according to the CPS.

Supplementary Table S1C. Body mass index and muscle related factors

		ALD (97)	HBV (133)	HCV (82)	MASLD (298)	PBC/AIH (73)	Others (128)	P value
BMI (kg/m^2)	n	97	133	82	298	73	128	<0.00001
	Me(Q1~Q3)	22.30 (20.06~24.82)	23.01 (20.79~25.24)	22.53 (19.67~24.44)	26.95 (24.37~29.33)	21.8 (19.02~23.82)	22.42 (20.15~24.99)	
BMI>23 kg/m^2	<23	54 (55.7)	66 (49.6)	50 (61.0)	46 (15.4)	45 (61.6)	76 (59.4)	<0.00001
	>23	43 (44.3)	67 (50.4)	32 (39.0)	252 (84.6)	28 (38.4)	52 (40.6)	
BMI Grade	Low (<20)	14 (14.4)	13 (9.8)	14 (17.1)	4 (1.3)	16 (21.9)	20 (15.6)	<0.00001
	Normal (20-25)	61 (62.9)	84 (63.2)	51 (62.2)	81 (27.2)	48 (65.8)	76 (59.4)	
	Obesity I (25-30)	16 (16.5)	29 (21.8)	15 (18.3)	146 (49.0)	9 (12.3)	28 (21.9)	
	Obesity II (30-35)	4 (4.1)	6 (4.5)	2 (2.4)	48 (16.1)	0 (0.0)	4 (3.1)	
	Obesity III (35-40)	1 (1.0)	1 (0.8)	0 (0.0)	13 (4.4)	0 (0.0)	0 (0.0)	
	Obesity IV (>40)	1 (1.0)	0 (0.0)	0 (0.0)	6 (2.0)	0 (0.0)	0 (0.0)	
Grip	n	97	131	81	298	73	128	<0.00001

strength (GS) (Kg)	Me(Q1~Q3)	23.5 (17.5~31.3)	20.8 (15.6~27.2)	16.0 (9.0~24.8)	21.1 (15.0~30.3)	17.8 (12.2~24.0)	19.8 (14.1~25.5)	
GS Low/Normal	Low	52 (53.6)	70 (53.4)	63 (76.8)	140 (47.0)	39 (53.4)	75 (58.6)	0.00020
	Normal	45 (46.4)	61 (46.6)	19 (23.2)	158 (53.0)	34 (46.6)	53 (41.4)	
Sarcopenia Index (SI)	n	97	132	82	298	73	128	0.00004
	Me(Q1~Q3)	69.2 (58.2~87.9)	76.3 (64.5~92.1)	66.8 (57.4~77.3)	75.3 (64.9~87.5)	69.7 (57.7~83.2)	76.6 (66.9~89.5)	
CBMM	n	97	132	82	298	73	128	<0.00001
	Me(Q1~Q3)	37.73 (34.03~42.70)	34.66 (29.30~43.04)	32.25 (26.88~39.23)	36.89 (31.95~45.22)	29.57 (25.99~34.87)	33.41 (29.52~40.68)	
CBMM Sp	Normal	47 (48.5)	97 (73.5)	37 (45.1)	264 (88.6)	40 (54.8)	86 (67.2)	<0.00001
	Sp	50 (51.5)	35 (26.5)	45 (54.9)	34 (11.4)	33 (45.2)	42 (32.8)	
dGFR	n	97	133	82	298	73	128	<0.00001
	Me(Q1~Q3)	12.10 (-5.00~24.22)	0.00 (-11.62~8.82)	9.60 (1.20~19.00)	-0.85 (-10.60~9.30)	3.50 (-10.92~14.07)	-0.30 (-14.50~9.45)	
SARC-F	n	97	131	81	298	73	128	0.00504
	Me(Q1~Q3)	1 (0~3)	0 (0~1.8)	1 (0~4)	1 (0~2)	1 (0~2)	0 (0~2)	
SARC-F Sp	Normal	75 (77.3)	113 (86.3)	60 (74.1)	262 (87.9)	63 (86.3)	108 (84.4)	0.01905
	Sp	22 (22.7)	18 (13.7)	21 (25.9)	36 (12.1)	10 (13.7)	20 (15.6)	

The abbreviations are as follows; body mass index (BMI), BMI group (BMIG), grip strength (GS), sarcopenia index (SI), calculated body muscle mass (CBMM), difference of CrGFR and cysGFR (dGFR). GS low was defined as <18 kg of mean grip strength in woman and <28 kg in man. CBMM sarcopenia (Sp) was defined as <27.9 in woman and <39.7 in man. SARC-F sarcopenia (Sp) was defined as > 4 points.

Supplementary Table S2A. The relation with liver stiffness (LS) >10 kPa and body mass index and grip strength in all patients.

LS>10 kPa		<10	>10	P
BMI (kg/m^2)	n	622	189	0.00071
	Me(Q1~Q3)	23.59 (21.14~26.62)	24.91 (21.93~28.50)	
BMI>23	<23	273 (43.9)	64 (33.9)	0.01429
	>23	349 (56.1)	125 (66.1)	
BMI G	Low (<20)	65 (10.5)	16 (8.5)	0.00248
	N (20-25)	322 (51.8)	79 (41.8)	
	I (25-30)	183 (29.4)	60 (31.7)	
	II (30-35)	38 (6.1)	26 (13.8)	
	III (35-40)	11 (1.8)	4 (2.1)	
	IV (>40)	3 (0.5)	4 (2.1)	
Grip strength (GS) Low/Normal	Low	324 (52.3)	115 (60.8)	0.03800
	Normal	296 (47.7)	74 (39.2)	
GS (kg)	n	619	189	0.99446
	Me(Q1~Q3)	20.5 (14.6~27.7)	21.3 (14.3~28.3)	

The abbreviations are as follows; body mass index (BMI), BMI group (BMIG), grip strength (GS). GS low was defined as <18 kg of mean grip strength in woman and <28 kg in man. n is number of patients. Me (Q1-Q3) is median (first quartile to the third quartile). P-values were calculated using the chi-square test or the Mann-Whitney test.

Supplementary Table S2B. The relation with liver stiffness >10 kPa and clinical factors in all patients.

LS>10 kPa		<10	>10	P
Sex	Women	364 (58.5)	78 (41.3)	0.00003
	Men	258 (41.5)	111 (58.7)	
Age	n	622	189	0.01182
	Me(Q1~Q3)	64.0 (52.0~74.0)	68.0 (59.0~73.0)	
Total Bilirubin (mg/dl)	n	621	189	<0.00001
	Me(Q1~Q3)	0.70 (0.50~0.90)	0.90 (0.70~1.40)	
Albumin (g/dl)	n	620	189	<0.00001
	Me(Q1~Q3)	4.30 (4.00~4.50)	4.00 (3.50~4.23)	
Prothrombin time-INR	n	621	189	<0.00001
	Me(Q1~Q3)	0.98(0.94~1.03)	1.06 (1.00~1.13)	
Creatinine (Cr) (mg/dl)	n	621	189	0.20507
	Me(Q1~Q3)	0.75(0.64~0.87)	0.77 (0.64~0.95)	
CrGFR (ml/min/1,73m^2)	n	621	189	0.59063
	Me(Q1~Q3)	69.3 (58.3~81.1)	70.4 (57.4~83.7)	
Cystatin C (CysC) (mg/l)	n	621	189	<0.00001

	Me(Q1~Q3)	0.97(0.83~1.19)	1.13 (0.99~1.41)	
CysGFR (ml/min/1,73m^2)	n	621	189	<0.00001
	Me(Q1~Q3)	72.3(55.07~87.52)	59.2 (44.5~71.4)	
Platelet (X10^4/μl)	n	621	189	<0.00001
	Me(Q1~Q3)	20.7 (17.2~24.9)	15.3 (11.4~20.0)	
AST (U/l)	n	621	189	<0.00001
	Me(Q1~Q3)	32.0 (23.0~53.3)	66.0 (43.8~106.5)	
ALT (U/l)	n	621	189	<0.00001
	Me(Q1~Q3)	35.0(20.0~70.0)	55.0 (34.7~107.2)	

Normal range is the follows; total bilirubin; 0.3-1.2 mg/dl, albumin, albumin; 3.8-5.2 g/dl, prothrombin time - international normalized ration (INR); 1.8-1.2, Creatinine (Cr); 0.47-0.79 mg/dl in woman and 0.61-1.04 mg/dl in man. Cr-estimated glomerular filtration rate (CrGRR): >60 ml/min/1,73m^2, cystatin C (CysC); 0.56-0.87 mg/l in woman and 0.63-0.95 mg/l in man, CysC-eGFR; >60 ml/min/1,73m^2, platelet; 13-36.9 X10^4/μl in woman and 13.1-36.2 X10^4/μl in man. Aspartate aminotransferase (AST); 10-40 U/l, Alanine aminotransferase (ALT); 5-40 U/l.

Supplementary Table S2C. The relation with liver stiffness >10 kPa and non-invasive liver fibrosis tests, muscle markers in all patients.

LS>10kPa		<10	>10	P
APRI	n	621	189	<0.00001
	Me(Q1~Q3)	0.5 (0.4~0.9)	1.6 (1.0~2.6)	

APRI 1.5	Normal	552 (88.9)	88 (46.6)	<0.00001
	High	69 (11.1)	101 (53.4)	
FIB-3	n	621	189	<0.00001
	Me(Q1~Q3)	1.5 (0.3~3.0)	5.1 (3.8~6.9)	
FIB-3 1.89	Normal	355 (57.2)	13 (6.9)	<0.00001
	High	266 (42.8)	176 (93.1)	
FIB-4	n	621	189	<0.00001
	Me(Q1~Q3)	1.7 (1.1~2.6)	4.1 (2.6~6.7)	
FIB-4 1.3	Normal	196 (31.6)	9 (4.8)	<0.00001
	High	425 (68.4)	180 (95.2)	
dGFR	n	621	189	<0.00001
	Me(Q1~Q3)	-0.400 (-12.900~9.400)	11.400 (-0.725~22.175)	
Sarcopenia Index (SI)	n	621	189	<0.00001
	Me(Q1~Q3)	75.8 (64.6~90.6)	66.7 (55.8~78.6)	
CBMM	n	621	189	0.38426
	Me(Q1~Q3)	35.02 (29.90~42.20)	35.98(30.142~43.2)	
CBMM Sarcopenia (Sp)	Normal	461 (74.2)	110 (58.2)	0.00002
	Sp	160 (25.8)	79 (41.8)	
SARC-F	n	619	189	0.00006
	Me(Q1~Q3)	0 (0~2)	1 (0~3)	

SARC-F Sp	Normal	537 (86.8)	144 (76.2)	0.00048
	Sp	82 (13.2)	45 (23.8)	

The abbreviations are as follows; fibrosis-4 index (FIB-4), AST to platelet ratio (APRI), fibrosis- 3 index (FIB-3), sarcopenia index (SI), calculated body muscle mass (CBMM), difference of CrGFR and cysGFR (dGFR). The APRI cutoff value was set at 1.5, and the high group was defined as follows. The FIB-3 cutoff value was set at 1.89. The FIB-4 cutoff value was set at 1.3. CBMM sarcopenia (Sp) was defined as <27.9 in woman and <39.7 in man. SARC-F sarcopenia (Sp) was defined as > 4 points.

Supplementary Table S3. Progression of LS related to mSpOb and dGOb score.

MASLD: LS kPa		0-10	10-15	15-20	20-	P
mSpOb	0	70 (31.4)	8 (22.2)	3 (17.6)	1 (4.5)	0.01853
	1	117 (52.5)	17 (47.2)	8 (47.1)	13 (59.1)	
	2	36 (16.1)	11 (30.6)	6 (35.3)	8 (36.4)	
dGOb	0	82 (36.8)	7 (19.4)	3 (17.6)	1 (4.5)	0.00007
	1	98 (43.9)	20 (55.6)	9 (52.9)	7 (31.8)	
	2	43 (19.3)	9 (25.0)	5 (29.4)	14 (63.6)	
BMI 26/27	N	126 (56.5)	18 (50.0)	6 (35.3)	7 (31.8)	0.06256
	mOb	97 (43.5)	18 (50.0)	11 (64.7)	15 (68.2)	
GS 16/31	N	131 (58.7)	15 (41.7)	8 (47.1)	8 (36.4)	0.06284
	mSp	92 (41.3)	21 (58.3)	9 (52.9)	14 (63.6)	
dGFR 1.14/-0.76	N	136 (61.0)	16 (44.4)	9 (52.9)	2 (9.1)	0.00003
	SpdG	87 (39.0)	20 (55.6)	8 (47.1)	20 (90.9)	

mSpOb score and dGOb graded to 0, 1 and 2. BMI graded to mOb (>26 kg/m^2 in woman and >27 kg/m^2 in man) and normal (N). GS graded to mSp (<16 kg of mean grip strength in woman and <31 kg in man) and normal (N). dGFR graded to SpdG (<1.14 of dGFR in woman and <-0.76 in man) and normal (N). P-values were calculated using the chi-square test.

Supplementary Table S4. SpOb, mSpOb, SIOb and dGOb were contributed to LS >10kPa in MASLD.

set	item	P	Odds ratio	95%CI lower	95%CI upper
A	APRI 1.5	0.01167	2.394	1.215	4.718
A	FIB-3 1.89	0.00050	4.436	1.918	10.257
A	FIB-4 1.3	0.16356	1.913	0.768	4.766
A	SpOb score	0.04315	1.642	1.015	2.654
B	APRI 1.5	0.01482	2.332	1.180	4.609
B	FIB-3 1.89	0.00055	4.345	1.888	10.001
B	FIB-4 1.3	0.14787	1.963	0.787	4.895
B	mSpOb score	0.01193	1.737	1.129	2.672
C	APRI 1.5	0.01472	2.340	1.182	4.633
C	FIB-3 1.89	0.00080	4.190	1.814	9.677
C	FIB-4 1.3	0.12173	2.060	0.825	5.143
C	SIOb score	0.00931	1.711	1.142	2.566
D	APRI 1.5	0.02185	2.235	1.124	4.443
D	FIB-3 1.89	0.00087	4.126	1.791	9.508
D	FIB-4 1.3	0.08274	2.268	0.899	5.718
D	dGOb score	0.00169	1.913	1.276	2.868

E	APRI 1.5	0.01308	2.368	1.199	4.679
E	FIB-3 1.89	0.00059	4.323	1.875	9.970
E	FIB-4 1.3	0.06368	2.420	0.951	6.157
E	BMI26/27	0.01459	2.110	1.159	3.842
F	APRI 1.5	0.01339	2.338	1.193	4.582
F	FIB-3 1.89	0.00027	4.724	2.050	10.888
F	FIB-4 1.3	0.25150	1.717	0.682	4.323
F	GS16/31	0.26091	1.403	0.777	2.532
G	APRI 1.5	0.01362	2.338	1.191	4.590
G	FIB-3 1.89	0.00038	4.563	1.975	10.543
G	FIB-4 1.3	0.23412	1.744	0.698	4.357
G	SI67/84	0.14230	1.555	0.862	2.805
H	APRI 1.5	0.02174	2.218	1.123	4.379
H	FIB-3 1.89	0.00043	4.480	1.944	10.326
H	FIB-4 1.3	0.19849	1.823	0.730	4.552
H	dGFR1.14/-0.76	0.01963	2.019	1.119	3.643

In each set, factors contributing to LS >10kPa were analyzed by multiple logistic regression analysis was performed. Set were APRI (>1.5 group), FIB-3 (>1.89 group), FIB-4 (>1.3 group) and osteosarcopenia related factor (SpOb score (A), mSpOb score (B), SIOb score (C), dGOb score (D), BMI (>26 in woman and >27 man group) (E), GS (<16 kg in woman and <31 in man) (F), SI (<67 in woman and <84 in man) (G) and dGFR (>1,14 in woman and >-076 in man) (H).

Supplementary Table S5. Difference between ALD and MASLD after propensity score matched

Propensity Score matched		ALD	MASLD	P
Sex	Woman	17 (23.0)	21 (28.4)	0.45165
	Man	57 (77.0)	53 (71.6)	
Age	n	74	74	0.53172
	Me(Q1~Q3)	63.0 (53.0~72.0)	63.0 (57.0~71.0)	
Total Bilirubin (mg/dl)	n	74	74	0.95549
	Me(Q1~Q3)	0.85 (0.60~1.40)	0.80 (0.60~1.20)	
Albumin (g/dl)	n	74	74	0.57763
	Me(Q1~Q3)	4.20 (3.90~4.40)	4.10 (3.90~4.40)	
Prothrombin time-INR	n	74	74	0.88103
	Me(Q1~Q3)	1.03 (0.95~1.10)	1.01 (0.95~1.12)	
Platelet ($X10^4/\mu l$)	n	74	74	0.60196
	Me(Q1~Q3)	18.10 (13.30~24.00)	19.30 (14.80~23.20)	
AST (U/l)	n	74	74	0.99694
	Me(Q1~Q3)	40.0 (27.0~78.0)	41.5 (29.0~65.0)	

ALT (U/l)	n	74	74	0.03081
	Me(Q1~Q3)	31.5 (20.0~67.0)	47.5 (33.0~71.0)	
LS (kPa)	n	74	74	0.60864
	Me(Q1~Q3)	6.5 (4.3~12.3)	7.4 (4.8~13.7)	
BMI 26/27	N	62 (83.8)	43 (58.1)	0.00058
	Ob	12 (16.2)	31 (41.9)	
GS 16/31	N	33 (44.6)	36 (48.6)	0.62108
	Sp	41 (55.4)	38 (51.4)	
dGFR 1.14/-0.76	N	29 (39.2)	28 (37.8)	0.86586
	SpdG	45 (60.8)	46 (62.2)	
dGOb	0	22 (29.7)	19 (25.7)	0.00125
	1	47 (63.5)	33 (44.6)	
	2	5 (6.8)	22 (29.7)	
mSpOb	0	26 (35.1)	22 (29.7)	0.02129
	1	43 (58.1)	35 (47.3)	
	2	5 (6.8)	17 (23.0)	
LS>10 kPa	<10	47 (63.5)	48 (64.9)	0.86387
	>10	27 (36.5)	26 (35.1)	

Sex, age, total bilirubin, albumin, prothrombin time INR, platelet, AST, ALT and LS were covariates. Obesity (Ob) is >26 of BMI in woman and >27 in man. Low grip strength (Sp) is <16 kg in woman and <31 kg in man. High dGFR (SpdG) is >1.14 in woman and >-0.76 in man. dGOb and mSpOb were score. P value was calculated by Mann-Whitney test or chi-square test.

Supplementary Table S6A. Background of the group whose body composition was examined by CT in MASLD

Body Composition		CT	None	P
Sex	Women	62 (71.3)	117 (55.5)	0.01127
	Men	25 (28.7)	94 (44.5)	
Age	n	87	211	0.00085
	Me(Q1~Q3)	65.0 (58.0~72.5)	59.0 (49.0~69.0)	
LS (kPa)	n	87	211	0.01169
	Me(Q1~Q3)	6.8 (5.05~13.3)	6.1 (4.4~8.8)	
APRI	n	87	211	0.33243
	Me(Q1~Q3)	0.8 (0.5~1.3)	0.7 (0.5~1.3)	
APRI 1.5	Normal	70 (80.5)	174 (82.5)	0.68293
	High	17 (19.5)	37 (17.5)	
FIB-3	n	87	211	0.18695
	Me(Q1~Q3)	2.4 (1.1~4.4)	1.9 (0.8~3.9)	
FIB-3 1.89	Normal	35 (40.2)	101 (47.9)	0.22880
	High	52 (59.8)	110 (52.1)	
FIB-4	n	87	211	0.00507
	Me(Q1~Q3)	2.0 (1.3~3.5)	1.7 (1.1~2.6)	
FIB-4 1.3	Normal	20 (23.0)	71 (33.6)	0.06926
	High	67 (77.0)	140 (66.4)	
ALBI	n	87	211	0.00020

	Me(Q1~Q3)	-2.8699 (-3.06547~-2.5819)	-2.9901 (-3.1576~-2.7870)	
ALBIG	1	65 (74.7)	193 (91.5)	0.00010
	2	19 (21.8)	17 (8.1)	
	3	3 (3.4)	1 (0.5)	
mSpOb score	0	14 (16.1)	68 (32.2)	0.01311
	1	50 (57.5)	105 (49.8)	
	2	23 (26.4)	38 (18.0)	
dGOb score	0	21 (24.1)	72 (34.1)	0.18835
	1	41 (47.1)	93 (44.1)	
	2	25 (28.7)	46 (21.8)	

High group was >1.5 of APRI, >1,89 of FIB-3, >1,3 of FIB-4. ALBIG is AIBI grade. P value calculated by Mann-Whitney test or chi-square test.

Supplementary Table S6B. Comparison of body composition by mSpOb score in MASLD

Sex		Women				Men				ALL			
mSpOb		0	1	2	P	0	1	2	P	0	1	2	p
SM	n	9	37	16	0.41277	5	13	7		14	50	23	

	Me(Q1~Q3)	90.1 (73.9~110.5)	94.5 (82.0~112.6)	98.8 (94.0~109.8)		151.1 (148.5~167.1)	146.8 (126.2~160.3)	155.3 (148.8~165.8)	0.41455	111.9 (87.6~149.1)	103.5 (84.0~137.4)	109.1 (95.9~152.5)	0.35406
IMAT	n	9	37	16	0.16000	5	13	7	0.15337	14	50	23	0.16692
	Me(Q1~Q3)	10.9 (7.0~15.2)	9.3 (5.9~15.2)	15.6 (7.7~20.5)		7.7 (6.5~12.9)	4.4 (1.5~11.0)	7.1 (4.8~9.5)		9.3 (6.9~12.2)	8.1 (5.2~12.5)	12.8 (5.8~19.3)	
VAT	n	9	37	16	0.01925	5	13	7	0.16513	14	50	23	0.04433
	Me(Q1~Q3)	83.0 (40.1~147.0)	134.6 (93.7~210.5)	201.5 (134.3~223.3)		220.1 (204.8~256.7)	171.2 (72.3~259.1)	246.7 (214.3~330.7)		162.9 (51.6~216.5)	143.6 (90.3~220.4)	211.2 (158.3~237.6)	
SAT	n	9	36	16	0.06183	5	13	7	0.05639	14	49	23	0.00598
	Me(Q1~Q3)	156.4 (133.3~185.2)	167.3 (126.1~207.5)	195.2 (184.4~233.0)		147.4 (135.0~155.3)	113.7 (89.6~142.6)	201.3 (151.1~245.4)		151.7 (142.4~181.8)	145.8 (101.2~205.1)	195.8 (177.8~233.0)	
MA	n	9	37	16	0.07492	5	13	7	0.45005	14	50	23	0.48221
	Me(Q1~Q3)	27.7 (24.9~33.6)	26.5 (23.6~33.7)	22.7 (19.3~29.5)		37.2 (34.6~37.9)	38.6 (32.2~41.4)	39.7 (37.1~43.4)		32.8 (26.4~36.0)	30.4 (24.0~35.9)	25.2 (20.3~37.6)	
SMI	n	9	37	16	0.02770	5	13	7		14	50	23	

	Me(Q1~Q3)	37.2 (30.6~41.4)	39.5 (36.8~47.1)	43.6 (40.4~47.7)		54.4 (50.4~56.1)	52.6 (49.1~57.7)	55.1 (54.3~61.3)	0.29083	41.8 (36.5~52.2)	44.7 (37.5~52.1)	44.9 (41.4~55.1)	0.16773
SMIL	Low	5 (55.6)	14 (37.8)	1 (6.3)	0.02112	0 (0.0)	1 (7.7)	1 (14.3)	0.66623	5 (35.7)	15 (30.0)	2 (8.7)	0.09326
	Normal	4 (44.4)	23 (62.2)	15 (93.8)		5 (100.0)	12 (92.3)	6 (85.7)		9 (64.3)	35 (70.0)	21 (91.3)	
Sarcopenia JSH	Sp	2 (22.2)	12 (32.4)	1 (6.3)	0.12262	0 (0.0)	1 (7.7)	1 (14.3)	0.66623	2 (14.3)	13 (26.0)	2 (8.7)	0.19269
	N	7 (77.8)	25 (67.6)	15 (93.8)		5 (100.0)	12 (92.3)	6 (85.7)		12 (85.7)	37 (74.0)	21 (91.3)	

Body composition: SM: skeletal muscle (cm^2), IMAT; intra-muscle adipose tissue (cm^2), VAT; visceral adipose tissue (cm^2), SAT; subcutaneous adipose tissue (cm^2), MA; muscle attenuation (HU), SMI; SM (cm^2)/$Hight^2$ (m^2), SMIL; SMI low group <38 cm^2/m^2 of SMI in woman and <42 cm^2/m^2 in man. Sarcopenia JSH; <18 kg of grip strength in woman and <28 kg in man and SMIL by sarcopenia criteria of The Japan society of Hepatology (JSH). P value was evaluated by Kruskal-Wallis test.

Supplementary Table S6C. Comparison of body composition by dGOb score in MASLD

Sex		Women				Men				All			
dGOb		0	1	2	P	0	1	2	P	0	1	2	P
SM	n	13	31	18		8	10	7		21	41	25	

	Me(Q1~Q3)	82.8 (73.5~91.5)	99.3 (84.3~115.3)	102.2 (94.5~111.2)	0.00481	146.7 (134.4~149.7)	155.6 (151.1~168.8)	155.3 (142.1~169.6)	0.23181	95.4 (76.4~140.8)	105.2 (85.8~139.8)	109.1 (98.0~149.1)	0.13428
IMAT	n	13	31	18	0.29938	8	10	7	0.13567	21	41	25	0.29711
	Me(Q1~Q3)	8.2 (6.0~11.9)	10.7 (6.4~19.8)	15.2 (5.9~19.8)		5.3 (1.6~11.7)	8.6 (6.3~11.8)	4.6 (3.3~5.6)		7.8 (5.2~11.9)	10.1 (6.3~17.6)	8.6 (4.7~17.0)	
VAT	n	13	31	18	0.00211	8	10	7	0.31685	21	41	25	0.01104
	Me(Q1~Q3)	84.2 (45.7~108.4)	173.1 (105.5~229.5)	146.1 (120.2~217.2)		209.5 (101.6~231.8)	266.7 (200.5~296.4)	216.5 (159.0~282.5)		101.1 (50.2~207.7)	204.4 (106.8~239.2)	168.6 (131.5~222.7)	
SAT	n	12	31	18	0.00007	8	10	7	0.07125	20	41	25	0.00000
	Me(Q1~Q3)	96.7 (83.3~154.2)	185.0 (148.3~206.1)	202.1 (183.8~241.5)		108.1 (90.7~144.9)	142.6 (99.7~166.9)	201.3 (137.6~245.2)		100.0 (89.7~149.7)	166.9 (138.8~203.9)	201.3 (179.8~243.8)	
MA	n	13	31	18	0.70454	8	10	7	0.34040	21	41	25	0.46481
	Me(Q1~Q3)	25.5 (23.1~34.0)	27.0 (20.4~31.6)	24.0 (22.5~31.8)		37.5 (32.6~44.4)	37.0 (30.6~41.1)	38.8 (38.2~40.3)		32.3 (25.1~35.9)	28.6 (22.8~35.5)	30.5 (23.2~37.9)	
SMI	n	13	31	18		8	10	7		21	41	25	

	Me(Q1~Q3)	36.5 (30.6~38.9)	42.7 (37.3~47.5)	44.1 (39.5~48.6)	0.00199	50.4 (48.6~53.1)	56.3 (54.4~60.5)	54.1 (52.9~58.2)	0.15676	42.5 (33.7~49.7)	44.4 (37.9~53.1)	44.9 (40.8~55.2)	0.06536
SMIL	Low	10 (76.9)	9 (29.0)	1 (5.6)	0.00013	0 (0.0)	1 (10.0)	1 (14.3)	0.56956	10 (47.6)	10 (24.4)	2 (8.0)	0.00859
	Normal	3 (23.1)	22 (71.0)	17 (94.4)		8 (100.0)	9 (90.0)	6 (85.7)		11 (52.4)	31 (75.6)	23 (92.0)	
Sarcopenia JSH	Sp	7 (53.8)	8 (25.8)	0 (0.0)	0.00245	0 (0.0)	1 (10.0)	1 (14.3)	0.56956	7 (33.3)	9 (22.0)	1 (4.0)	0.03814
	N	6 (46.2)	23 (74.2)	18 (100.0)		8 (100.0)	9 (90.0)	6 (85.7)		14 (66.7)	32 (78.0)	24 (96.0)	

Supplementary Table S7. Comparison of body composition between mSpOb and non-mSpOb in MASLD

Sex		Women			Man		
mSpOb		Non- mSpOb	mSpOb	p	Non-mSpOb	mSpOb	p
SM	n	46	16	0.19809	18	7	0.30353
	Me(Q1~Q3)	93.0 (81.4~111.6)	98.8 (94.0~109.8)		149.7 (138.9~164.8)	155.3 (148.8~165.8)	
IMAT	n	46	16	0.07158	18	7	0.62826
	Me(Q1~Q3)	9.5 (6.1~14.7)	15.6 (7.7~20.5)		6.1 (2.2~10.8)	7.1 (4.8~9.5)	
VAT	n	46	16	0.03239	18	7	0.10223
	Me(Q1~Q3)	121.1 (86.5~207.2)	201.5 (134.3~223.3)		209.5 (130.9~249.3)	246.7 (214.3~330.7)	
SAT	n	45	16	0.02171	18	7	0.03415
	Me(Q1~Q3)	164.4 (126.1~205.1)	195.2 (184.4~233.0)		128.0 (92.2~151.4)	201.3 (151.1~245.4)	
MA	n	46	16	0.02431	18	7	0.39682
	Me(Q1~Q3)	26.7 (23.6~33.7)	22.7 (19.3~29.5)		37.8 (32.3~40.6)	39.7 (37.1~43.4)	
SMI	n	46	16	0.06431	18	7	0.13026

	Me(Q1~Q3)	39.0 (36.1~45.8)	43.6 (40.4~47.7)		53.3 (49.6~57.5)	55.1 (54.3~61.3)	
SMIL	Low	19 (41.3)	1 (6.3)	0.00978	1 (5.6)	1 (14.3)	0.49000
	Normal	27 (58.7)	15 (93.8)		17 (94.4)	6 (85.7)	
Sarcopenia-JSH	Sp	14 (30.4)	1 (6.3)	0.08751	1 (5.6)	1 (14.3)	0.49000
	N	32 (69.6)	15 (93.8)		17 (94.4)	6 (85.7)	

Non-mSoOb is the group excluding mSpOb from the total.
